# The WSD-type wax ester synthase is widely conserved in streptophytes and crucial for floral organ formation under high humidity in land plants

**DOI:** 10.1007/s10265-025-01628-6

**Published:** 2025-04-01

**Authors:** Takashi Nobusawa, Yuko Sasaki-Sekimoto, Hiroyuki Ohta, Makoto Kusaba

**Affiliations:** 1 Graduate School of Integrated Sciences for Life, Hiroshima University, 1-4-3, Kagamiyama, Higashi-Hiroshima, 739-8526 Japan; 2 Phytolipid Technologies Inc., 4259-3, Nagatsuta, Midori-ku, Yokohama, 226-8510 Japan

**Keywords:** Adaptation to land environments, Ambient humidity, Arabidopsis, Cuticular wax, *Klebsormidium*, Plant evolution

## Abstract

Cuticular waxes are a complex mixture of long-chain aliphatic compounds, including alkanes, aldehydes, alcohols, ketones, and esters, that cover the outer surface of most terrestrial plants. While cuticular waxes play a pivotal role in plant adaptation to various environmental stresses, the specific roles of individual molecular species, particularly wax esters (WEs), remain poorly understood. In this study, we investigate the evolution and function of wax ester biosynthesis in land plants, focusing on the WSD (wax synthase/DGAT) enzyme family. We demonstrate that the ancestral origin of WSD enzymes traces back to streptophyte algae, specifically *Klebsormidium nitens* (KnWSD1). Our findings provide evidence that KnWSD1 functions as a monofunctional WSD catalyzing WE formation without producing triacylglycerols (TAGs). By generating Arabidopsis mutants with multiple *WSD* gene knockouts, we uncover a crucial role for WEs in supporting floral organ development under high humidity. Our results reveal that WEs are essential for floral organ development and provide new insights into their evolutionary significance in plant adaptation to terrestrial environments.

**Supplementary Information:**

The online version contains supplementary material available at 10.1007/s10265-025-01628-6.

## Introduction

The primary aerial surfaces of land plants are covered by a protective layer consisting of a cutin polyester matrix embedded with cuticular waxes (Yeats and Rose 2013). Among their various functions, cuticular waxes are particularly important for providing resistance to abiotic stresses, especially desiccation (Kosma et al. 2009; Shepherd and Griffiths 2006). Cuticular waxes in land plants primarily consist of very-long-chain fatty acids (VLCFAs) with carbon chains exceeding 20 atoms, as well as VLCFA-derived compounds such as alkanes, aldehydes, alcohols, and wax esters (Kunst and Samuels 2009). Wax esters (WEs), neutral lipids formed by the esterification of a fatty acyl group with a fatty alcohol, represent a minor fraction of the cuticular wax composition. For instance, in Arabidopsis inflorescence stems, WEs account for only up to 3% of total waxes (Li et al. 2008). Furthermore, the presence or absence of WEs in epicuticular waxes has no direct correlation with leaf desiccation tolerance, leaving the biological significance of WEs largely unclear (Patwari et al. 2019).

In addition to plants, WEs are also found in bacteria and animals (Petronikolou and Nair 2018). Industrial production of WEs primarily relies on extraction from low-yield sources such as jojoba oil. Alternatively, synthetic methods are used, which require high energy consumption and complex chemical reactions (Domergue and Miklaszewska 2022). Consequently, there has been significant interest in developing biological production systems using WE-synthesizing enzymes in plants or other platforms (Domergue and Miklaszewska 2022; Petronikolou and Nair 2018).

WE biosynthesis involves the enzymatic reaction between an activated fatty acid (via CoA or ACP) and a fatty alcohol, catalyzed by wax synthases (WS), which belong to three families: diacyl-glycerol O-acyltransferase (DGAT) 1, DGAT2, and WS/DGAT (WSD) (Röttig and Steinbüchel 2013). The first characterized WS enzyme was a DGAT1-type enzyme from jojoba (Lardizabal et al. 2000; Metz et al. 2000). In plants, functional analysis of WS homologs is still limited. For example, Rice OsWS1 has been implicated in cuticular wax synthesis (Xia et al. 2015). In Arabidopsis, AtWSD1, a homolog of the bacterial enzyme first identified in *Acinetobacter baylyi* (AbWSD1), has been established as a major WS (Kalscheuer and Steinbüchel 2003; Li et al. 2008; Stöveken et al. 2005). The WSD-type enzymes attract much attention, and their crystal structures were solved for *Marinobacter aquaeolei* MaWSD1 (Petronikolou and Nair 2018), followed by AbWSD1 (Vollheyde et al. 2022).

**Fig. 1:**
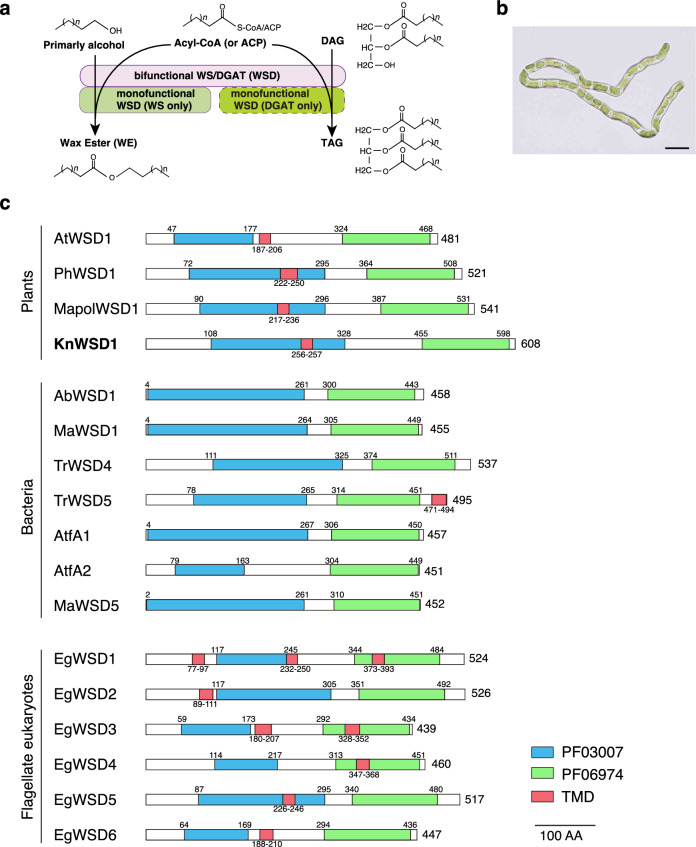
Semi-aquatic alga *Klebsormidium* has a conserved WSD-type wax synthase. **a** The biosynthetic pathways of WEs and TAGs catalyzed by WSD enzymes. Monofunctional WSD enzymes exhibit either WS or DGAT activity, whereas bifunctional WSD enzymes catalyze both processes. **b** A microscopic image of the *K. nitens* NIES-2285 strain. Bar, 20 µm. **c** Comparison of domain structures among well-characterized WSD homologs in plants, bacteria, and flagellate eukaryotes (e.g., *Euglena*). The PF03007 wax ester synthase-like Acyl-CoA acyltransferase domain and the PF06974 WS/DGAT C-terminal domain are highlighted in blue and green boxes, respectively. Transmembrane domains (TMDs) predicted by the Phobius program are indicated in red boxes. Numbers to the right indicate the total amino acid (AA) length, while numbers above or below each domain represent the start and end positions

WSD-type enzymes, extensively studied in bacteria, include both monofunctional enzymes that exclusively synthesize WEs and bifunctional enzymes capable of producing both WEs and triacylglycerols (TAGs) (Vollheyde et al. 2022) (Fig. 1a). In plants, only a few WSD homologs have undergone thorough biochemical characterization. For example, *Petunia hybrida* PhWSD1 is monofunctional, synthesizing only WEs (King et al. 2007), whereas Arabidopsis AtWSD1 is bifunctional, producing WEs and trace amounts of TAGs (Li et al. 2008). The roles and functional diversification of other WSD homologs in Arabidopsis, where ten homologs are present, remain unclear (Li et al. 2008; Patwari et al. 2019).

Recent research suggests that the acquisition of WSD homologs played a key role in the early adaptation of plants to terrestrial environments. For instance, in the *Marchantia polymorpha*, the knockout of *MpWSD1* results in reduced WE levels with impaired osmotic tolerance, highlighting the evolutionary significance of WEs (Keyl et al. 2024). However, research on WSD homologs in algae and knowledge regarding the origins of WSD acquisition in plants remain insufficient.

To understand the evolutionary adaptation of plants to terrestrial environments, studies focusing on extant streptophyte algae are considered valuable (Vries and Archibald 2018). Among these, *Klebsormidium nitens*, a member of Klebsormidiophyceae (Fig. 1b), is an alga capable of adapting to moist terrestrial environments. Gene sets specific to land plants, which are absent in other algae, have been identified starting from the evolutionary stage of *Klebsormidium* (Hori et al. 2014). While *K. nitens* lacks a cuticle layer, it possesses a primitive structure of glycoprotein frameworks associated with triacylglycerols (Kondo et al. 2016). In contrast, gametophores of *Physcomitrium* (*Physcomitrella*) *patens* develop a cuticle composed of land plant-type cutin, which is thought to involve the contribution of *sn*-2 glycerol-3-phosphate acyltransferase (*sn*-2 GPAT) acquired during this evolutionary period (Lee et al. 2020). Both *P. patens* and *M. polymorpha* synthesize primary alcohols and WEs (Keyl et al. 2024), suggesting that the acquisition of surface lipid biosynthetic pathways from *Klebsormidium* to bryophytes played a key role in the terrestrialization of plants.

In our previous studies, Kondo et al. (2016) identified a gene in *K. nitens* (KnWSD1) with sequence similarity to WSD, suggesting that the acquisition of WSD homologs dates back to the Klebsormidiophyceae. Although *K. nitens* retains a primitive surface lipid layer dominated by TAGs, neither WEs nor their key precursors, primary alcohols, have been detected in its surface lipids. The absence of primary alcohols is likely due to the lack of fatty acyl-CoA reductase (FAR), which is essential for their biosynthesis in land plants. Consequently, the absence of WEs in *K. nitens* raises questions about the molecular function of KnWSD1 (Kondo et al. 2016).

In this study, we demonstrate that *Klebsormidium* WSD, a key enzyme in WE biosynthesis in land plants, functions as a monofunctional WE synthase. KnWSD1 catalyzes the esterification of alcohols and fatty acids without producing TAGs. To further investigate the functional roles of WEs in surface lipids, we generated multiple Arabidopsis *WSD* mutants expressed in flowers. Our observations uncovered a novel role for WEs in supporting normal floral organ development under high-humidity conditions.

## Materials and methods

### Plant materials and growth conditions

Seeds of *wsd1-1* (SALK_067714, Li et al. 2008), *wsd6-2* (SALK_130459; Patwari et al. 2019), and *fop1-3/wsd11* (SALK_137481; Takeda et al. 2013) were obtained from the Arabidopsis Biological Resource Center (ABRC). All plants were transgenic *Arabidopsis thaliana* (L.) Heynh. (Arabidopsis) mutants with a Columbia-0 wild-type background. Seeds were sown on Jiffy-7 substrates and cultivated under a 16-h light/8-h dark cycle at 23 °C. Relative humidity (RH) was controlled at 40–50% or approximately 100% using an ITBOX-SH incubator (ITPLANTS, Japan).

### Plasmid construction and transformation

The coding sequence of *KnWSD1*, along with a linker and *Venus*, was PCR-amplified from *K. nitens* cDNA and cloned into the pHANNIBAL vector, which contains a CaMV35S promoter and OCS terminator, using the In-Fusion HD cloning kit (Takara Bio, Japan). It was subsequently subcloned into a pART27 binary vector using the NotI restriction sites. To generate *ProWSD1:GUS*, a 3-kb upstream region of WSD1 was PCR-amplified and cloned into a modified Gateway pENTR vector containing an MCS, GUS, and NOS terminator. For expressing *KnWSD1* in the *wsd* quintuple mutant, a DNA fragment containing 1122 bp of the *FIDDLEHEAD* (*FDH*) promoter region (Efremova et al. 2004) was PCR-amplified and cloned into a modified Gateway pENTR vector containing an MCS and NOS terminator. The codon-optimized *KnWSD1* sequence for Arabidopsis was then cloned into this vector. The Gateway vectors were subsequently subcloned into a pGWB601 binary vector using the Gateway LR Clonase enzyme mix (Invitrogen, USA). To generate multiple knockout mutants of Arabidopsis *WSDs*, we employed the CRISPR/Cas9 system. Guide RNA (gRNA) sequences—5′-CGGAAGCTGTGACCGTGGCT-3′ (for *WSD2*) and 5′-GTCTCTCAAGATATTTGAGC-3′ (for *WSD3*)—were cloned and assembled into the pENTR_AtU6gRNA2 vector as described previously (Nobusawa et al. 2021; Ito et al. 2023). The resulting construct with the two gRNAs was subcloned into the pGWB601_AtRPS5A-Cas9 binary vector (Ito et al. 2023; Nobusawa et al. 2021) using the Gateway LR Clonase enzyme mix (Invitrogen) and introduced into the *wsd1* mutant, which was then screened to isolate the *wsd1;2;3* triple mutant. The *wsd* quintuple mutant was generated by crossing the *wsd1;2;3* triple mutant with the *wsd6;11* mutant. Transformation of Arabidopsis was performed via the floral-dip method using the *Agrobacterium* strain EHA-105. For optimized expression in yeast, the coding sequence of *KnWSD1* was codon-optimized for *Saccharomyces cerevisiae* and cloned into the pYES2.1 vector (Invitrogen). The *Venus* gene in pYES2.1 was introduced previously in a similar manner (Nobusawa et al. 2017).

### In vitro enzyme assay conditions

The *S. cerevisiae* strain and methods for culture, transformation, enzyme isolation, confirmation of enzyme expression by Western blotting, and the in vitro enzyme assay were as previously described (Nobusawa et al. 2017), with the following modifications for the enzyme assay: 4.80 µM [1-14C]palmitoyl-CoA (50 mCi mmol⁻^1^, PerkinElmer, USA) was used as the acyl donor, and 3.75 mM each of 1-octadecanol (TCI, Japan), 1,2-dipalmitoyl-sn-glycerol (TCI), phytol (FUJIFILM Wako Pure Chemical Corporation, Japan), or stigmasterol (FUJIFILM Wako Pure Chemical Corporation) were used as acceptor substrates. Lipids were separated on a thin-layer chromatography (TLC) plate using a solvent system of hexane:ether:AcOH (40:10:1, v/v).

### Wax ester production in the yeast H1246 strain

For protein induction, cells were pre-cultured for 8 h in synthetic defined medium without uracil, containing 2% raffinose, at an initial OD600 of 0.2. Then, a 1/10 volume of 20% galactose solution and substrates (palmitic acid (TCI, 10% w/v in EtOH) and 1-octadecanol (Sigma-Aldrich, USA; 10% w/v in hot EtOH)) were added. After 48 h of cultivation at 180 rpm and 28 °C, cells were pelleted by centrifugation and lyophilized. Lipids were extracted using the Bligh and Dyer method and separated on a TLC plate using a solvent system of hexane:diethyl ether:acetic acid (40:10:1, v/v).

### Lipid quantification and qualification analysis

For the yeast experiment, TLC spots corresponding to esters were scraped from the plate and dissolved in diethyl ether. For surface lipid analysis in Arabidopsis, leaves were immersed in chloroform at room temperature for 30 s, and 100 nmol of C24-alkane was added as an internal standard. Leaf area was measured using Fiji (ImageJ) v2.9.0/1.53t. Qualitative analysis was performed using an Agilent 7890A GC system equipped with TOFMS (JMS-T100GCv, JEOL, Japan) and a DB-1HT column (30 m, 0.25 mm i.d., 0.10 µm film thickness; Agilent, USA), with helium as the carrier gas at a constant flow of 20 cm/s. The injection temperature was set to 390 °C. The temperature program began at 120 °C, increased by 15 °C per minute to 240 °C, then by 5 °C per minute to 290 °C, and finally by 15 °C per minute to 390 °C, where it was held for 15 min. Quantitative analysis was conducted using a GC-FID system (GC-8860, Agilent), with the detector temperature set to 350 °C. The column, flow rate, and temperature program were identical to those used for the GC–MS analysis, except nitrogen was used as the carrier gas.

### In vitro recrystallization

Extracted cuticular wax was recrystallized as described by Jetter and Riederer (1994). Total wax extracted from Arabidopsis inflorescence stems was dissolved in ethyl acetate (10 mg/mL) and recrystallized on aluminum foil discs in a solvent vapor-saturated glass chamber. Specimens were then coated with gold using an Auto Fine Coater (DII-29010SCTR, JEOL) and observed under a scanning electron microscope (SEM; JCM-6000, JEOL) in high-vacuum mode. To quantify the shape of wax crystals, the outlines of each crystal were traced and analyzed for circularity and solidity using Fiji (ImageJ) v2.9.0/1.53t.

### Microscopic observations

Subcellular localization was observed using an LSM780 confocal laser scanning microscope (Carl Zeiss, Germany). Maximum intensity projections were generated from acquired z-stacked images. GUS staining was performed as previously described (Nobusawa et al. 2013). Alexander staining and Aniline Blue staining were conducted following the protocols of Alexander (1969) and Lu (2011), respectively.

### RNA extraction and reverse transcription

For RT-PCR analysis, total RNA was extracted using TRI Reagent (Molecular Research Center, Inc., USA). First-strand cDNA was synthesized from 500 ng of total RNA using the ReverTra Ace qPCR RT Master Mix (TOYOBO, Japan) following the manufacturer’s instructions. For transcriptome analysis, total RNA was extracted using ISOSPIN Plant RNA and analyzed using a Lasy-Seq platform (Clockmics Inc., Japan). The resulting reads were mapped to the reference Arabidopsis genome, with poly-A sequences trimmed during processing. Quantification analysis was performed using the Subio Platform (version 1.24.5853, Subio Inc., Japan).

Oligo DNAs used in this study are listed in Table S2.

## Results and discussion

### *Klebsormidium* possesses a monofunctional WSD

WSD (wax synthase/acyl-CoA acyltransferase) is conserved across land plants but is absent in chlorophyta, suggesting that it was acquired evolutionarily near the lineage of streptophyta such as *Klebsormidium* (Kong et al. 2020; Kondo et al. 2016). WSD enzymes with known activity all share a conserved structure with PF03007 at the N-terminus and PF06974 at the C-terminus (Fig. 1c; Fig. S1). WSD from *K. nitens* (KnWSD1) also exhibits this same domain architecture, suggesting a similar enzymatic function as other WSDs. While bacterial WSDs do not consistently predict transmembrane domains (TMDs), TMDs are universally predicted in WSDs from land plants, including KnWSD1. For instance, bacterial AbWSD1 localizes to the cytoplasmic membrane, lipid inclusions, and partially in the cytoplasm (Stöveken et al. 2005). By contrast, AtWSD1 localizes specifically to the endoplasmic reticulum (ER; Li et al. 2008), and AtWSD6 and AtWSD7 localize to ER and partially in Golgi (Patwari et al. 2019), consistent with the ER being the primary site for lipid synthesis in plants (Ohlrogge and Browse 1995). TMDs are also conserved in the six WSDs of secondary endosymbiotic algae *Euglena* (EgWSD1–6, Tomiyama et al. 2017), suggesting a potential relationship between TMDs and the development of intracellular membrane systems.

**Fig. 2:**
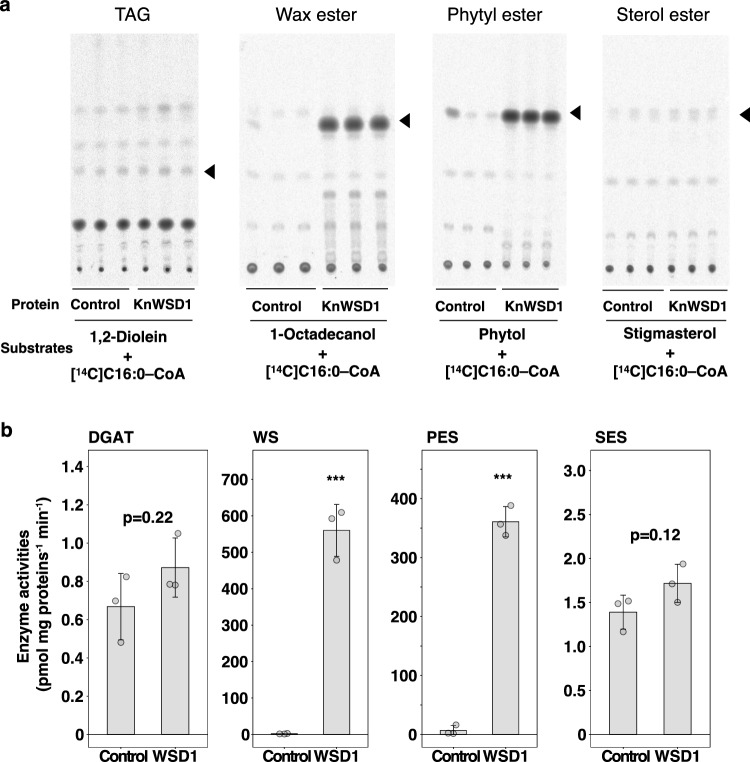
KnWSD1 exhibits wax ester (WE) synthetic activity in vitro. **a** Autoradiographic images of TLC plates after separating in vitro reactants, which include ^14^C-palmitoyl (C16:0)–CoA and various substrates, with crude protein extracts from *S. cerevisiae* containing the KnWSD1–V5–His enzyme. Triangles indicate the predicted main product positions. **b** Quantification of enzyme activity, with *** indicating *p* < 0.0001 by t-test

There are monofunctional WSDs that synthesize only wax esters (WE) or triacylglycerols (TAG), and bifunctional types with activity for both (Vollheyde et al. 2022). Although *Klebsormidium* does not synthesize WE, it secretes TAG extracellularly in a manner resembling a primitive surface lipids (Kondo et al. 2016). To investigate the substrate specificity of KnWSD1, we expressed the protein in *Saccharomyces cerevisiae* (Fig. S2). Using crude extracts, we tested DGAT and WE synthesis activities with radiolabeled C16:0-CoA as the donor substrate. While TAG synthesis activity was comparable to controls, significant WE synthesis activity was detected for KnWSD1 (Fig. 2).

Although WE has not been detected in *Klebsormidium*, it secretes minor amounts of phytyl esters (esters of phytol) and steryl esters (SE; esters of sterols) extracellularly, in contrast to the predominant secretion of TAG (Kondo et al. 2016). Using phytol and stigmasterol as alcohol substrates in in vitro assays, KnWSD1 produced phytyl esters but did not synthesize steryl esters (Fig. 2). These findings suggest that KnWSD1 is a monofunctional WSD that synthesizes esters from long-chain alcohols and acyl-CoA. While the bacterial *M. aquaeolei* MaWSD1 can also utilize substrates such as isoamyl alcohol and phenylethyl alcohol (Barney et al. 2015; Mancipe et al. 2022), KnWSD1’s capacity to use prenyl alcohols represents a novel functional feature. For bifunctional WSDs capable of synthesizing both WE and TAG, it is suggested that the ability of DAG to access the catalytic center is key (Santin et al. 2019; Vollheyde et al. 2022). In contrast, KnWSD1, which does not accommodate DAG, can still accommodate various long-chain alcohols, such as acyl- or prenyl-alcohols. This suggests that while the catalytic center of KnWSD1 is not sufficiently large to bind DAG, it retains enough flexibility to accept a range of alcohol substrates.

**Fig. 3:**
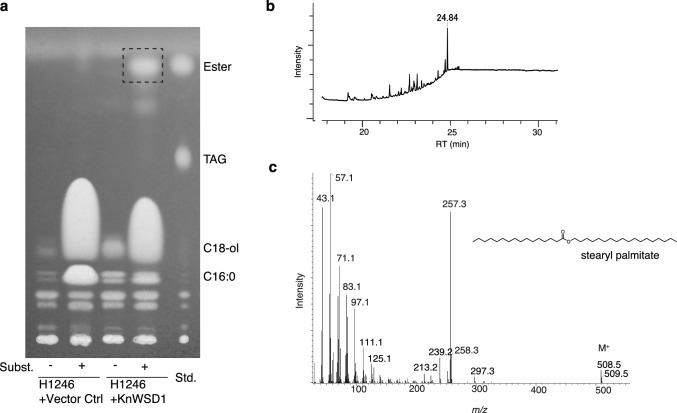
KnWSD1 produces WE in *S. cerevisiae* mutant lacking neutral lipid biosynthesis. **a** TLC image showing separated lipids extracted from *S. cerevisiae* H1246 strain expressing KnWSD1. The ester spot appears only in the presence of KnWSD1 with specific substrates. *Subst.* substrate, *Std.* standards, *ol* alcohol. **b**, **c** Generation of stearyl palmitate confirmed by GC-TOFMS. (B) A representative total ion chromatogram of lipids extracted from the region marked by the dotted box in (A). **c** The mass spectrum of the peak at a retention time of 24.84 min, closely matching that of stearyl palmitate (NIST database entry, https://webbook.nist.gov/cgi/cbook.cgi?ID=C2598994&Mask=200)

Next, we tested the ability of KnWSD1 to synthesize esters in the *S. cerevisiae* H1246 strain, which lacks neutral lipid biosynthesis (Sandager et al. 2002). When expressed alone, KnWSD1 did not produce esters in *S. cerevisiae* (Fig. 3). However, adding primary alcohols as substrates enabled the synthesis of WE (Fig. 3a, b). Despite the presence of endogenous DAG in yeast, TAG synthesis was not restored. These results confirm that KnWSD1 is a monofunctional WSD that utilizes long-chain alcohols as substrates.

**Fig. 4:**
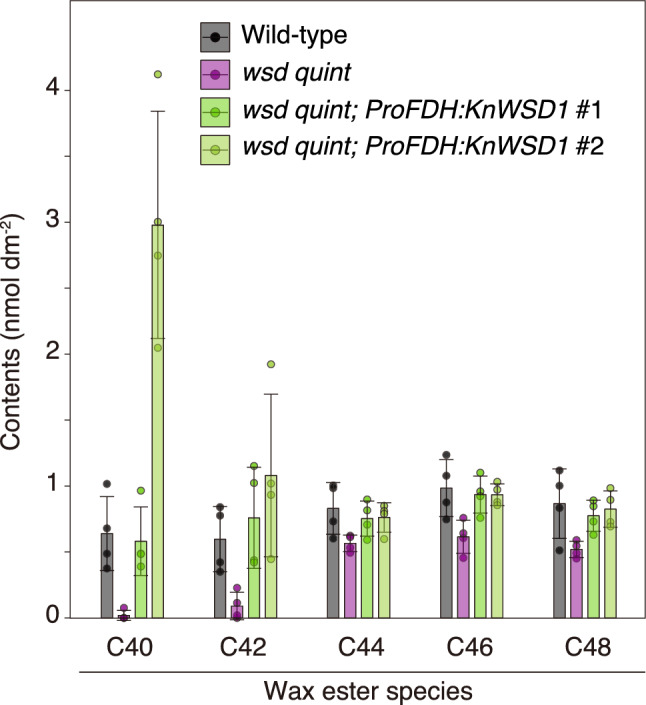
KnWSD1 biosynthesizes WEs *in planta*. Quantification of WE species in leaves of the Arabidopsis *wsd* quintuple mutant expressing *KnWSD1* under the *ProFDH* promoter. Values represent mean ± S.D. (*n* = 4 biological replicates)

To verify the WE synthase activity of KnWSD1 *in planta*, we expressed *KnWSD1* in the Arabidopsis *wsd quintuple* mutant (details of the *wsd* quintuple mutant are described later). In the *wsd* quintuple mutant, WE content in leaves was significantly reduced compared to the wild type, particularly in the C40 and C42 molecular species (Fig. 4). These reductions in WE were restored by the expression of *KnWSD1*, highlighting its function as a WE synthase. Furthermore, the expression of *KnWSD1* produced substantial amounts of WEs ranging from C40 to C48, suggesting that KnWSD1 has broad substrate specificity for acyl-chain length.

### KnWSD1 is an ER-localized protein

**Fig. 5:**
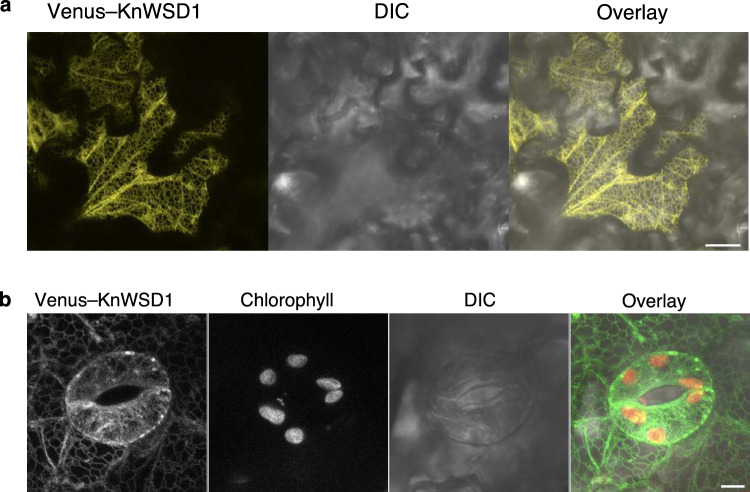
KnWSD1 localizes specifically to the ER. **a** Confocal microscopy of Arabidopsis epidermal pavement (**a**) and stomatal (**b**) cells expressing Venus–KnWSD1 shows a typical ER network-like pattern, with no chloroplast localization. Scale bars: 20 µm for (**a**), 5 µm for (**b**). DIC, differential interference contrast

While bacterial MaWSD1 localizes to the cytosol in plants, it produces different molecular species of WE when targeted to chloroplasts (Vollheyde et al. 2021). In Arabidopsis, the primary enzymes for phytyl ester synthesis, PES1 and PES2, localize to plastoglobules in the chloroplast (Lippold et al. 2012). Since KnWSD1 also utilizes phytol, we investigated its subcellular localization. Although a transformation system for *K. nitens* is not yet established, we expressed KnWSD1 in Arabidopsis for analysis. The Venus–KnWSD1 fusion protein localized specifically to the ER in Arabidopsis epidermal cells, and no chloroplast localization was observed (Fig. 5). This localization is consistent with the absence of chloroplast targeting signals and the presence of a KKXX motif at the C-terminus, which mediates ER retention in yeast, humans, and plants (Benghezal et al. 2000).

*K. nitens* also possesses PES homologs (Kondo et al. 2016), suggesting that KnWSD1 may not actively contribute to phytyl ester synthesis but instead esterifies trace phytol that leaks out of chloroplasts. Residual PE detected in Arabidopsis *pes1 pes2* mutants suggests the existence of additional PE-synthesizing enzymes (Lippold et al. 2012). Unlike wax biosynthesis genes typically expressed in the epidermis, AtWSD1 is ubiquitously expressed in Arabidopsis, possibly contributing to minor PE synthesis in mesophyll cells (Li et al. 2008).

Despite its evident WE synthesis activity, *K. nitens* lacks WEs and homologs of primary alcohol-synthesizing enzymes, such as fatty acyl-CoA reductase (FAR), found in land plants. Interestingly, WSD and FAR homologs are present in bryophytes such as *P. patens* and *M. polymorpha* (Keyl et al. 2024). In *P. patens*, WE is a major surface lipid component (Buda et al. 2013; Lee et al. 2020), and mutants lacking ABC transporters responsible for lipid secretion show desiccation sensitivity (Buda et al. 2013). The loss of osmotic stress tolerance in *MpWSD1* mutants (Keyl et al. 2024) highlights how early acquisition of WSD and WE synthesis capability may have facilitated the early adaptation to terrestrial environments of plants. Although *Klebsormidium* possesses flexible waxes primarily composed of TAGs that allow it to survive terrestrial environments, it may have been more advantageous to utilize solid waxes composed of long-chain lipids like WEs (wax esters), which solidify at room temperature, for better adaptation to terrestrial conditions.

### WEs alter wax crystal morphology

**Fig. 6:**
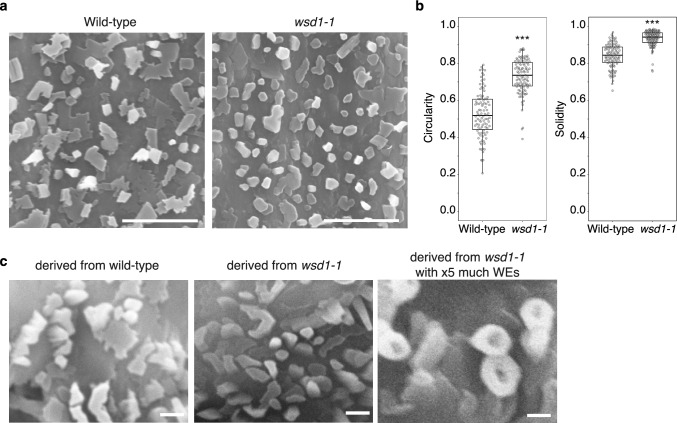
Wax ester (WE) alters the shape of epicuticular wax crystals. **a** SEM images of Arabidopsis inflorescence stem surfaces show that wax esters absence results in rounder crystal shapes. **b** Shape analysis of epicuticular wax crystals. Crystals from the wild-type and *wsd1-1* mutant were analyzed (*n* = 99 for wild-type,* n* = 101 for *wsd1-1*). Circularity measures how closely a shape resembles a perfect circle, with higher values indicating more circular and less elongated shapes. Solidity represents the ratio of the area of a shape to its convex hull, with higher values reflecting more compact and less irregular structures. Both circularity and solidity were significantly higher in the *wsd1-1* mutant compared to the wild-type (*p* < 0.001, Student’s *t*-test). Individual data points are shown on the box plot to display the distribution of all samples. The box represents the interquartile range (IQR), with the median indicated by a line inside the box. Whiskers extend to data within 1.5 × IQR. **c** Recrystallization of epicuticular wax in vitro. Surface lipids were extracted from plants with chloroform and recrystallized on aluminum. Lipids from wild-type, *wsd1* mutant, and *wsd1* mutant supplemented with five times the wild-type wax ester amount were used. Scale bars: 5 µm in (**a**) and 1 µm in (**c**)

While WEs became the predominant surface lipid in early land plants like bryophytes, only a limited number of seed plant species, such as *Copernicia sp.* (Carnauba) (Doan et al. 2017) and *Eucalyptus sp.* (Wirthensohn et al. 2000), use WEs as a major surface lipid. In the model plant Arabidopsis, WEs account for only up to 3% of the waxes on inflorescence stems (Li et al. 2008). On the other hand, the majority of plant species have waxes predominantly composed of alkanes. Although the physical and chemical properties of individual molecules that constitute plant surface lipids, particularly WEs, remain poorly understood, it is known that the morphology of wax crystals varies depending on the constituent lipid molecules. We discovered that the absence of WEs in Arabidopsis inflorescence stems resulted in wax crystals adopting a more rounded morphology (Fig. 6a, b). Waxes can recrystallize in vitro (Jetter and Riederer 1994), and waxes derived from both wild type and *wsd1* mutant plants recrystallized into the same shapes observed *in planta*. Interestingly, when WEs isolated from wild-type plants were added at a fivefold higher concentration to waxes derived from the *wsd1* mutant, the resulting crystals exhibited an even more rounded morphology (Fig. 6c). Tubular crystals formed by secondary alcohol-rich crystals (e.g., nonacosan-10-ol) are linked to superhydrophobicity (Barthlott and Neinhuis et al. 1997; Fleetwood et al. 2024). Thus, it is possible that trace components like WEs play a role of physical, chemical, or biological significance in wax crystal formation.

## *WSD* is essential for floral organ development under high humidity

A well-known function of surface lipids is to provide resistance to desiccation (Kosma et al. 2009; Shepherd and Griffiths 2006). In Arabidopsis, WEs are induced under drought conditions via WSD1 activity, and a *wsd1* mutant resulted in reduced drought tolerance due to increased stomatal density (Patwari et al. 2019). Non-stomatal water loss was found to be unchanged in *wsd1* mutants compared to wild type when stomata were kept closed (Fig. S3). While the biological significance of WEs and the roles of the 11 *WSD* genes in Arabidopsis are not fully understood, a *folded petal 1* (*fop1*) mutant that exhibits floral developmental abnormalities due to disrupted movement of petals during blooming has been identified, with *AtWSD11* being the responsible gene (Takeda et al. 2013). This gene is strongly expressed in pre-blooming petals (Takeda et al. 2013).

**Fig. 7:**
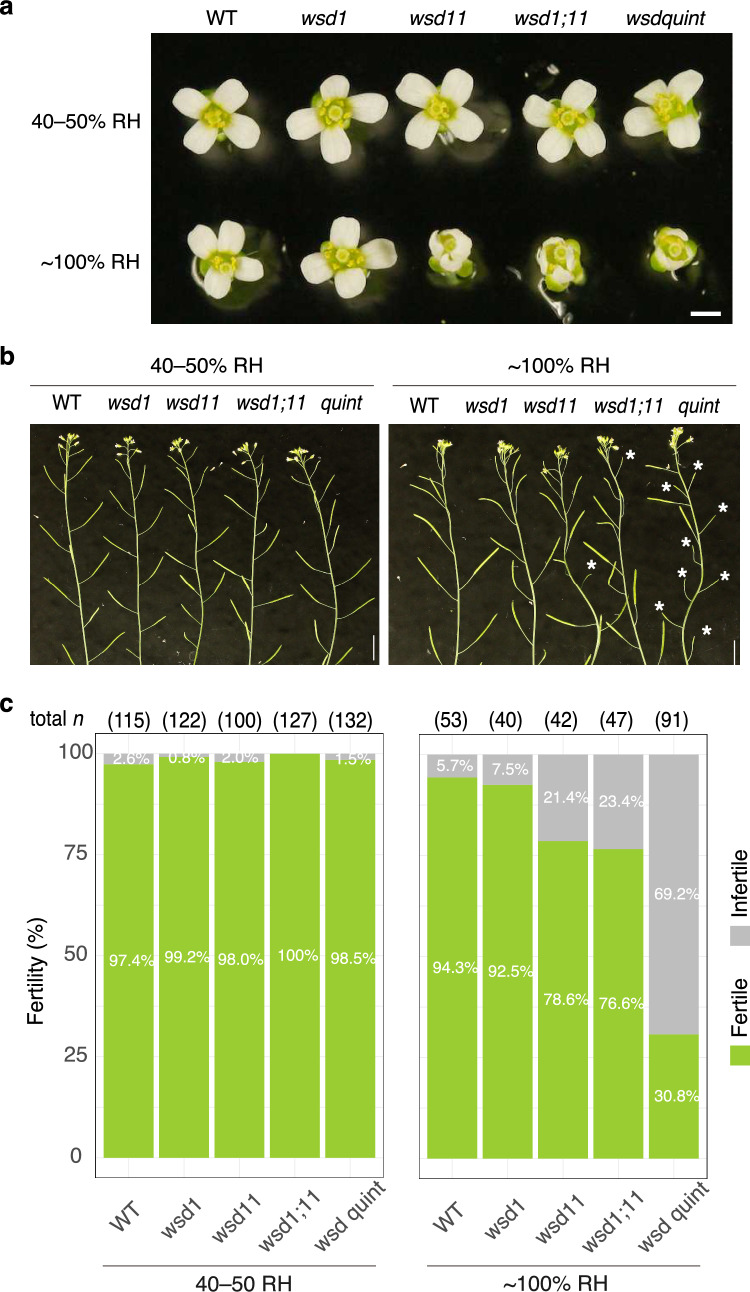
Wax ester (WE) is essential for floral organ formation under high humidity in Arabidopsis. **a** Flowers were grown under normal (40–50% RH) or high (near 100% RH) humidity conditions, then dissected and placed on a gel for imaging. Abnormal morphology was observed only in high RH conditions for *wsd* mutants. *quintuple* (quint) mutant refers to *wsd1;2;3;6;11* mutant. Scale bar, 1 mm. **b** Inflorescence of Arabidopsis plants grown for 10 days under varied RH conditions. Asterisks indicate flowers that failed to be pollinated. Scale bar, 1 cm. **c** Fertility rate (fertile: green, infertile: gray). Sample sizes (*n*) are shown above the bars

Interestingly, under our initial growth conditions (relative humidity of 40–50%), the floral phenotype of *fop1* mutants was not reproduced. However, under high-humidity conditions, we observed severe morphological abnormalities and reduced fertility, consistent with previous reports (Fig. 7; Takeda et al. 2013). Among the 11 *WSD* genes in Arabidopsis, *WSD1* is central for WE synthesis in inflorescence stems, as *wsd1* mutants completely lack WEs in this tissue (Li et al. 2008). Moreover, *WSD1* is strongly expressed in leaves and flowers (Li et al. 2008). By extending the promoter region from the previously reported 2 kb to 3 kb, we found that *WSD1* is expressed throughout flowers, not just in the stamen base as previously reported (Fig. S4).

To investigate further, we created a *wsd1;11* double mutant but observed no additive effects on floral morphology or fertility compared to *wsd11* mutants alone. RT-PCR analysis revealed that in addition to *WSD1* and *WSD11*, other homologs like *WSD2, WSD3,* and *WSD6* are expressed in flowers (Fig. S5). Further tissue-specific PCR showed distinct expression patterns for these genes in mature floral organs, suggesting specialized roles (Fig. S5). Using CRISPR/Cas9, we generated quintuple mutants of these five genes (Fig. S6). While floral morphology and fertility were unaffected under normal humidity conditions, high humidity caused severe fertility reductions (Fig. 7). However, given the minor contributions of *WSD2* and *WSD3* to WE synthesis in inflorescence stems, their roles in flowers are likely limited.

Aniline blue staining and Alexander staining revealed no abnormalities in pistil and pollen viability in the quintuple mutants (Fig. S7). Occasionally, siliques did develop in the quintuple mutants, suggesting that sterility was primarily due to disrupted contact between stamens and pistils caused by enhanced morphological abnormalities, preventing successful pollination (Fig. S8). These findings suggest that the sterility observed under high-humidity conditions results from enhanced *fop1*-like phenotypes.

### The role of WEs in floral development and their evolutionary perspective

The mechanism underlying WE biosynthesis in flowers appears to be widely conserved across plant species. For example, in *Petunia hybrida*, the monofunctional WSD enzyme PhWSD1, which exclusively synthesizes WEs, has been identified and is believed to function, at least, in the petals (King et al. 2007). Although analyzing surface lipids in plants with small flowers, such as Arabidopsis, poses challenges, it is possible that WEs are ubiquitously and actively synthesized in the flowers of many plant species. For instance, the genome of *Chrysanthemum seticuspe*, a model species for wild chrysanthemums, contains six WSD homologs (CsWSD1–6), among which *CsWSD1* shows pronounced expression in unopened flower buds and tubular flowers (Fig. S9). Tubular flowers exhibit a dense structure in which the stamens and pistils are tightly packed, likely creating a high-humidity microenvironment prior to blooming. Consistent with this, increased relative humidity at the center of flowers has been reported in *Oenothera cespitosa* (Arx et al. 2012). These observations suggest that WE biosynthesis may play a role in facilitating smooth contact between floral organs under high-humidity conditions during blooming.

Previously, it was reported that SAGL1, an F-box protein targeting CER3 (a key enzyme in alkane biosynthesis), exhibits expression changes in response to ambient humidity in Arabidopsis rosette leaves (Kim et al. 2019). To investigate the molecular-level impact of WEs on floral development under high-humidity conditions, we conducted a comprehensive analysis of differentially expressed genes using Lasy-Seq (Kamitani et al. 2019) in stage 12 and 13 flower buds of WT and quint mutants grown under both normal and high-humidity conditions. Unexpectedly, the number of differentially expressed genes was minimal, and we failed to identify any genes directly explaining the observed phenotypes (Table S1). Furthermore, the expression levels of *WSD* family genes remained unchanged under high-humidity conditions. These findings suggest that the morphological abnormalities caused by WE deficiency under high-humidity conditions are primarily mediated by mechanisms other than gene expression changes.

Interestingly, recent research on *M. polymorpha* reported that the loss of MpWSD1, which is responsible for WE biosynthesis, leads to reduced WE levels. While this does not affect growth under normal conditions, it significantly impairs growth under osmotic stress (Keyl et al. 2024). Although increased ambient humidity is unlikely to directly induce osmotic stress, WE deficiency might alter cellular responses related to water potential. However, the precise mechanisms remain to be elucidated.

## Conclusion

While WEs have become minor components of surface lipids in most land plants, our findings suggest that the ability to synthesize WEs, likely originating from streptophytes such as *Klebsormidium*, supported the early stages of plant terrestrialization. In angiosperms, WEs continue to play a critical role in floral organ formation and reproductive success under high-humidity conditions.

## Supplementary Information

Below is the link to the electronic supplementary material.

Supplementary file1 (PDF 10639 KB)

Supplementary file2 (XLSX 96 KB)

## Data Availability

The data that support the findings of this study are available from the corresponding author upon reasonable request.
