# Supplementary material for: The WSD-type wax ester synthase is widely conserved in streptophytes and crucial for floral organ formation under high humidity in land plants
Source: J Plant Res. 2025 Apr 1;138(3):497–509. doi: 10.1007/s10265-025-01628-6 (PMC12062176; doi:10.1007/s10265-025-01628-6)
Supplement: Supplementary file 1 — Supplementary file1 (PDF 10639 KB) [file 10265_2025_1628_MOESM1_ESM.pdf]

Supplementary information

The WSD-type wax ester synthase is widely conserved in streptophytes and crucial for floral organ formation under high humidity in land plants

Journal of Plant Research

Takashi Nobusawa[1]*, Yuko Sasaki-Sekimoto[2], Hiroyuki Ohta[2], Makoto Kusaba[1]

1 Graduate School of Integrated Sciences for Life, Hiroshima University, 1-4-3, Kagamiyama, Higashi-Hiroshima 739-8526, Japan
2 Phytolipid Technologies Inc., 4259-3, Nagatsuta, Midori-ku, Yokohama 226-8510, Japan

*For correspondence. E-mail nobusawa@hiroshima-u.ac.jp

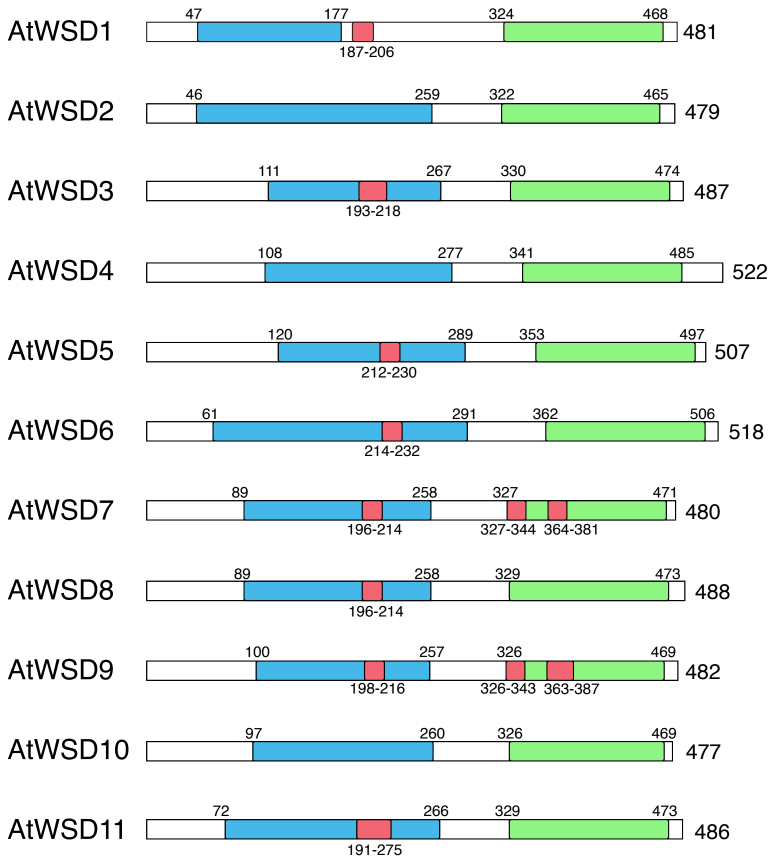

Fig. S1 Comparison of domain structures among Arabidopsis WSD homologs. The PF03007 wax ester synthase-like Acyl-CoA acyltransferase domain and the PF06974 WS/DGAT C-terminal domain are highlighted in blue and green boxes, respectively. Transmembrane domains (TMDs) predicted by the Phobius program are indicated in red boxes. Numbers to the right indicate the total amino acid (AA) length, while numbers above or below each domain represent the start and end positions.

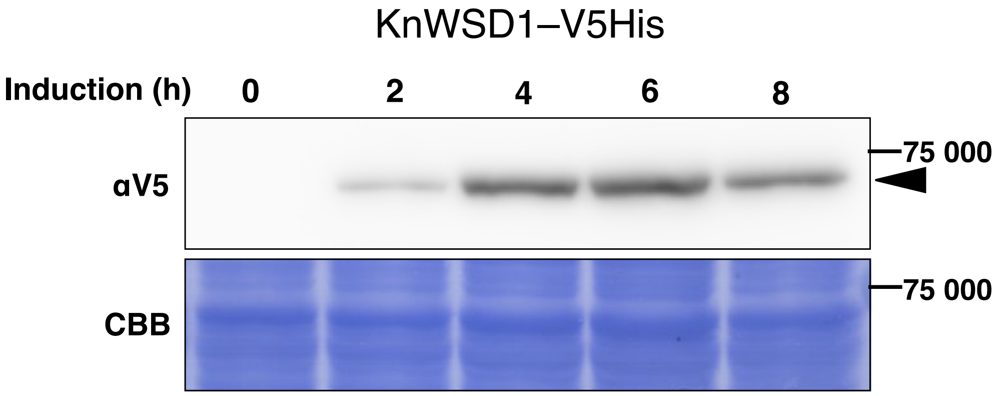

Fig. S2 Gel blot analysis of KnWSD1–V5His expressed in *S. cerevisiae*. Detection was performed using an anti-V5 antibody. The triangle indicates the estimated size of the KnWSD1–V5His protein. CBB, Coomassie Brilliant Blue staining.

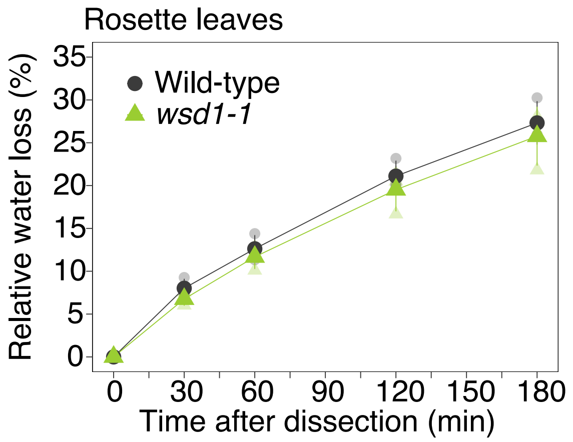

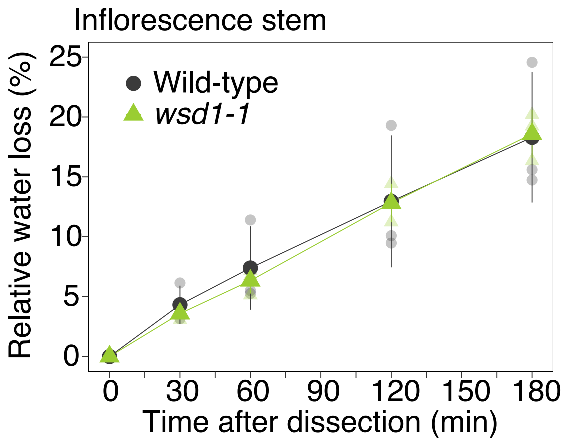

Fig. S3 Non-stomatal water loss assay in Arabidopsis. Stomata were closed by incubating the plants overnight in the dark, then rosette leaves or inflorescence stems were dissected and kept at RT. Fresh weight was measured over time while samples were maintained in the dark. Values indicate mean ± S.D. (n = 3 biological replicates). No statistical differences were observed between the wild-type and the *wsd1-1* mutant.

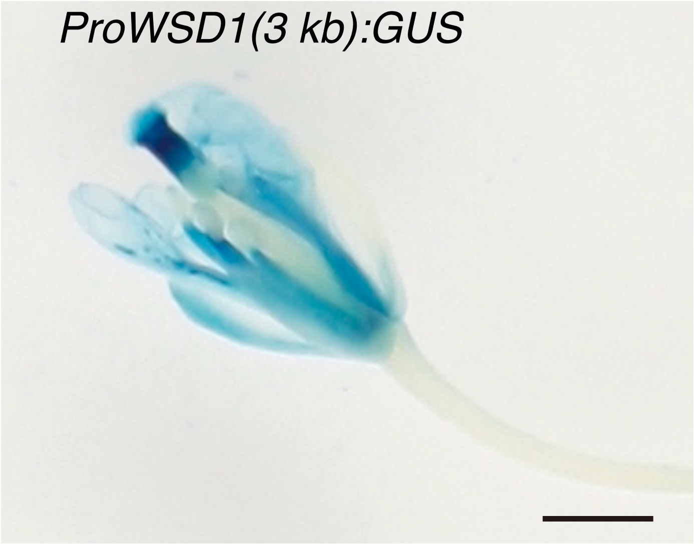

Fig. S4 Spatial expression patterns of *AtWSD1* using a 3-kb upstream promoter region. Similar patterns were observed in at least four independent transgenic lines. Scale bar, 1 mm.

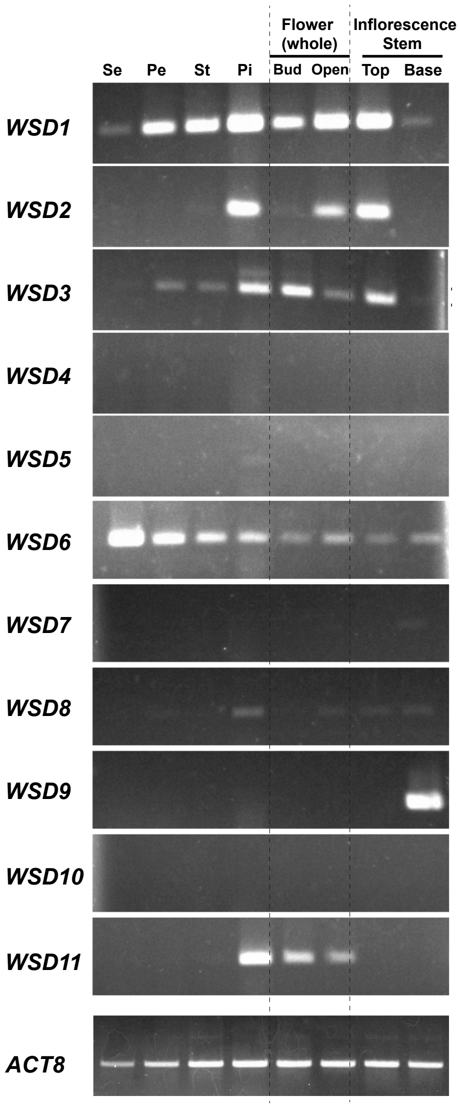

Fig. S5 RT-PCR analysis of 11 Arabidopsis *WSD* genes in flowers (buds and open flowers), inflorescence stems (Top: approximately 3 cm below the meristem; Base: 2–5 cm above the base), and each floral whorl at stages 13–14. Abbreviations: sepals (Se), petals (Pe), stamens (St), and pistils (Pi). *ACT8* was used as a control for quantitative consistency. Expression levels were assessed after 29 PCR cycles.

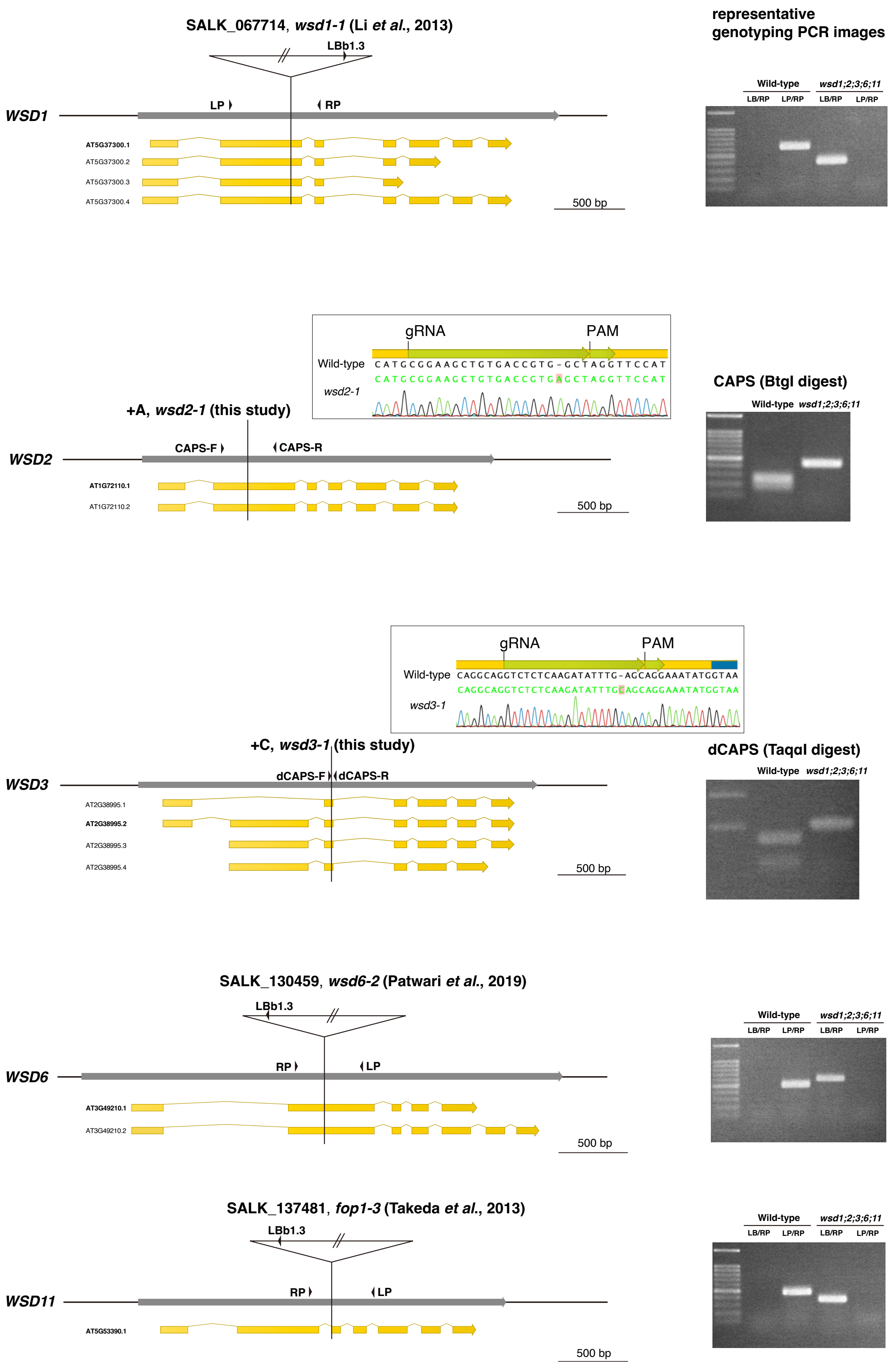

Fig. S6 Schematic model of the Arabidopsis *WSD1*, *2*, *3*, *6*, and *11* gene loci, with T-DNA insertion sites (for *wsd1-1*, *wsd6-2*, and *wsd11/fop1-3*) or mutated sequences generated by CRISPR/Cas9 (for *wsd2-1* and *wsd3-1*). Representative genotyping PCR images are shown on the right.

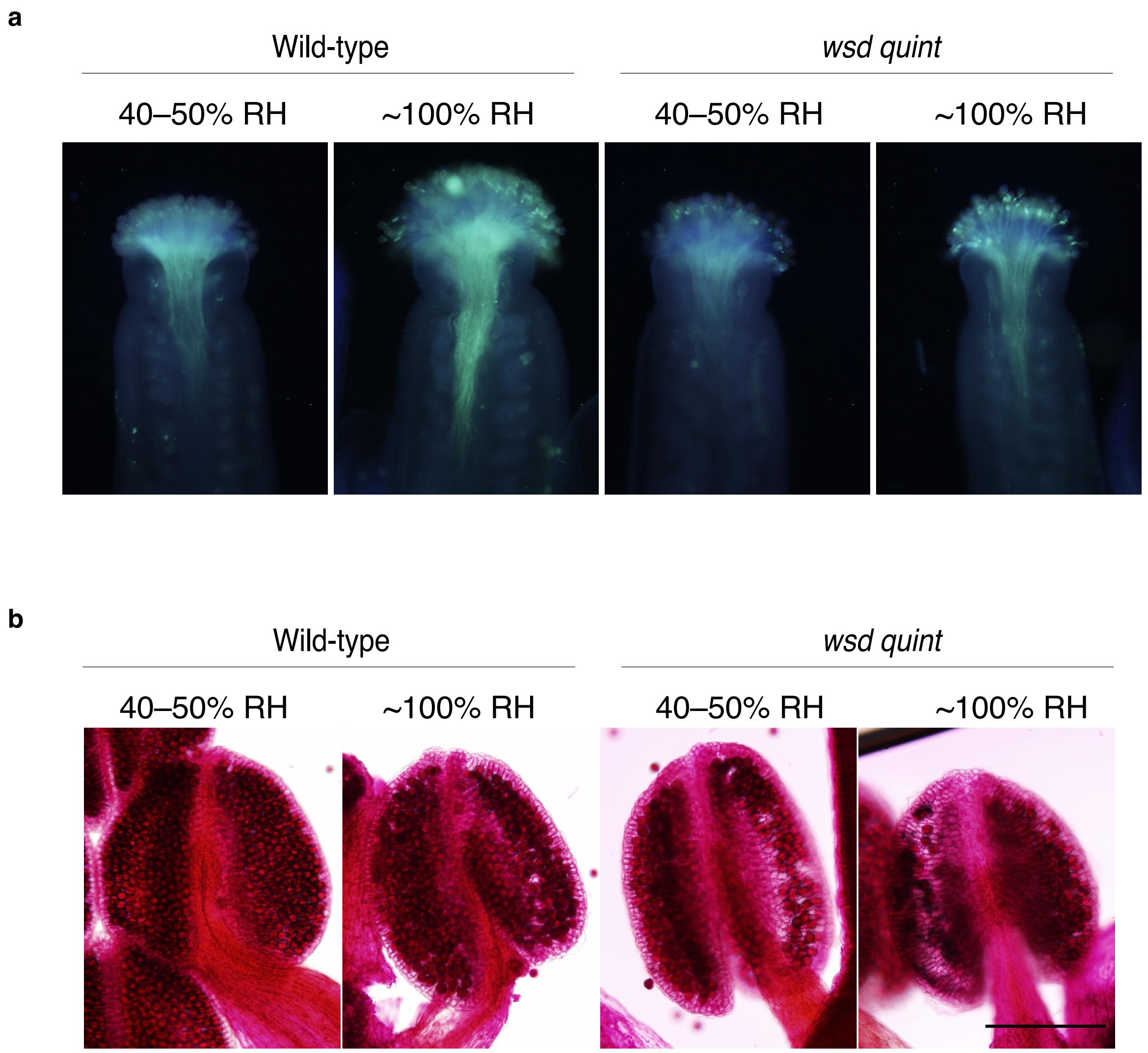

Fig. S7 Viability of pistils and pollen in the *wsd1;2;3;6;11* mutant under high RH conditions. (**a**) Wild-type pollen was manually pollinated onto pistils of both wild-type and *wsd1;2;3;6;11* mutant plants grown under high RH. Aniline blue staining of pollen tubes reveals no abnormalities. (**b**) Alexander staining of pollen shows that pollen viability is unaffected by the *wsd1;2;3;6;11* mutation or high RH. Scale bar, 200 μm.

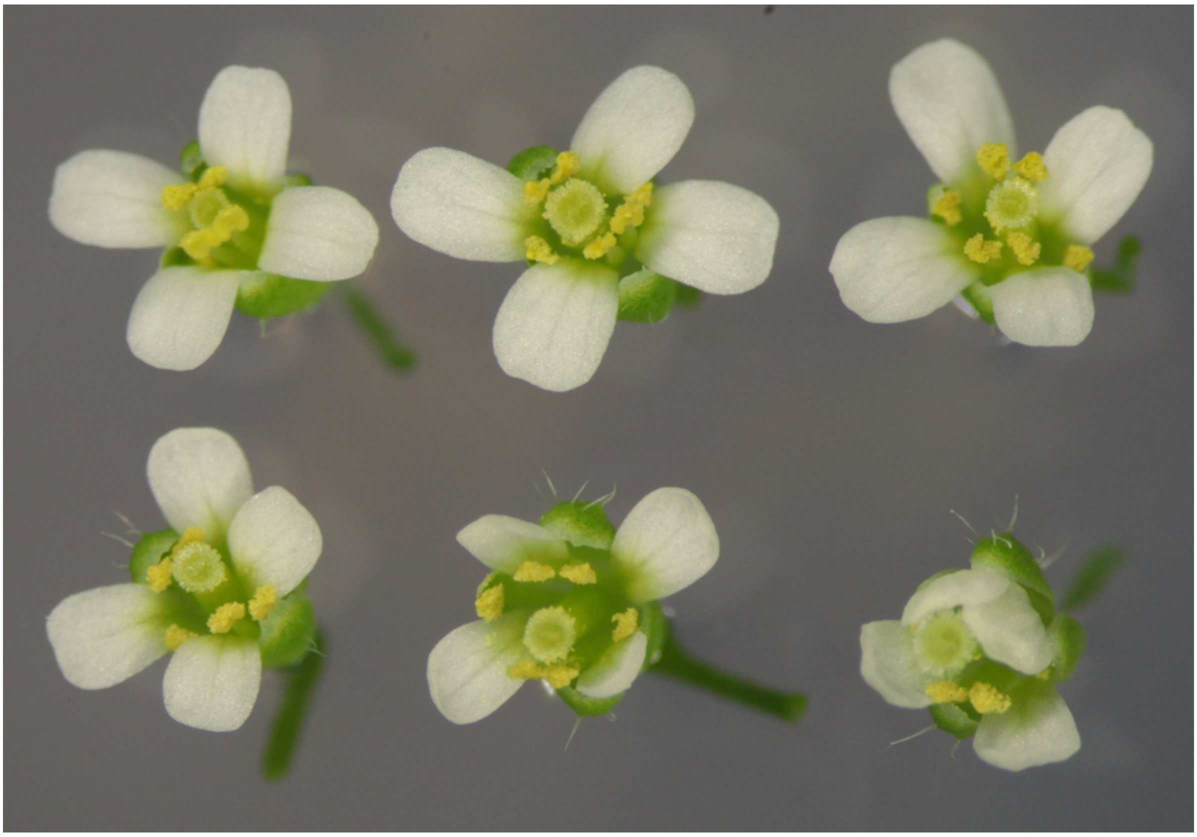

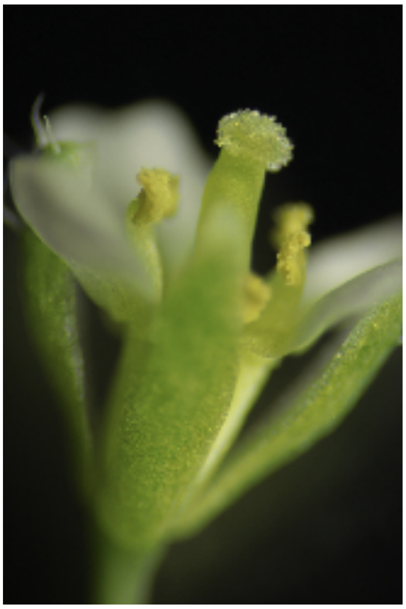

Fig. S8 Abnormal flower morphology in the *wsd1;2;3;6;11* quintuple mutant under high RH. Frequent failures in pistil-anther contact were observed.

a

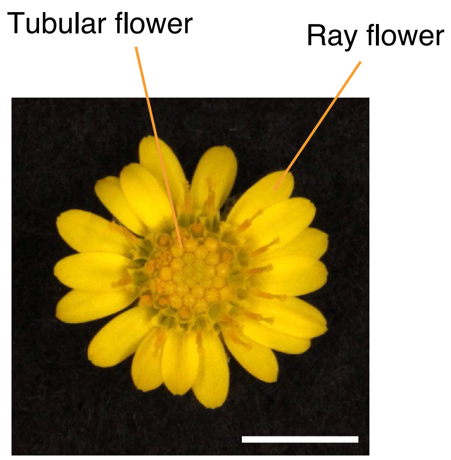

b

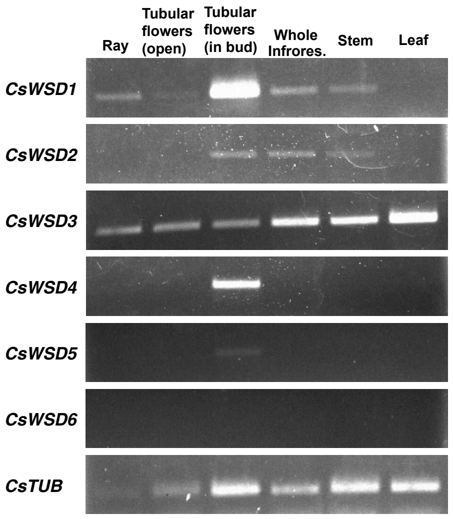

Fig. S9 Inflorescence of *Chrysanthemum seticuspe*. (**a**) Whole inflorescence of *C. seticuspe*. Scale bar, 0.5 cm. (**b**) RT-PCR analysis of *WSD* homologs in the *C. seticuspe* genome. Flower parts and conditions correspond to those annotated in (a). PCR cycles: 26 for *CsTUB* (tubulin) and 36 for *CsWSD1–6*.

Table S1 Differentially expressed genes under high humidity conditions.

#Table S1 is provided as a separate Excel file due to its large dataset. Please refer to the accompanying Table S1 file for detailed data.

Table S2 Primers used in this study.

DNA fragment (construct)	Forward primer (5′–3′) Reverse primer (5′–3′)
ProAtWSD1	CGGGATCCGTCGACCTAAGCCTTCTCTTGATTTCACTAC
(ProAtWSD1:GUS)	ATTCAATTAAAAGCTTTTGATGAGGAGATTATG
Venus	CACGCTCGAGGAATTATGGTGAGCAAGGGCGAG
(Pro35S:Venus–KnWSD1)	AGCGGAATTACCGGTCTTGTACAGCTCGTCCATG
KnWSD1_CDS	GGATCGACCCAAGGGATGCTTTTGTTGGGAGAGAC
(Pro35S:Venus–KnWSD1)	TAGAGGATCCAAGCTCTACGCCAACTTTTTGAAAG
linker	ACCGGTAATTCCGCTGA
(Pro35S:Venus–KnWSD1)	CCCTTGGGTCGATCCT
ProFDH	GGCCGCCATGGAATTAAGTCTACAACAATTAGCGTCCTCG
(ProFDH_modiviedENTR)	TAGAAAGCTTCTGCAGTAGGTTGATTATGTGAGTGAGATCTATA
KnWSD1_At_codon	GCGGAAAAAGAGCTTATGCTTCTCCTTGGAGAAACTTTTC
(ProFDH:KnWSD1optAt)	TTCGAGCTCTCTAGATCAAGCAAGTTTCTTGAAAGAGA
KnWSD1_Sc_codon	GCCCTTAAAAAAATGTTATTGCTTGGCGAGA
(KnWSD1optSc-V5His)	TCGAAGCTCGCCCTTTGCTAACTTCTTAAAAGATA

RT-PCR primers	(5′–3′)
RT-PCR__AtWSD1_F	GTCCCTACTACTTGCTTGTTCAC
RT-PCR__AtWSD1_R	TCAGCCGTTGATCCATCATATTTG
RT-PCR__AtWSD2_F	GCATACTGGTGACTGGTCATGG
RT-PCR__AtWSD2_R	CCCAAAGAATGATGGAACCTAGC
RT-PCR__AtWSD3_F	ATCCGTCTTCGTGGTACAGTATTC
RT-PCR__AtWSD3_R	CACATAGTAGATGAGGGTCTCG
RT-PCR__AtWSD4_F	CAACGATGTTGTACTTGGAGTAACC
RT-PCR__AtWSD4_R	TACTAGCCGCCACCTTGATTC
RT-PCR__AtWSD5_F	AGAGAAGATACGGAGAACAAGAAGAG
RT-PCR__AtWSD5_R	CACTAATGACCGTTGGATCGAC
RT-PCR__AtWSD6_F	AAAGAGAAAGACTAAGCAGCAAACC
RT-PCR__AtWSD6_R	GAGATGTCTAAAGGGACCGTCG
RT-PCR__AtWSD7_F	TGTCTTTGATTTCCCTCATGCTC
RT-PCR__AtWSD7_R	TCCTTCTTCGTGCCATACTGTC
RT-PCR__AtWSD8_F	GCGTTGGAGAGAGAGAATAAACATG
RT-PCR__AtWSD8_R	TTCACCGAAGGAATAGCAGTAGAC
RT-PCR__AtWSD9_F	ATTGGTTCTTGTGGTTTGTTCC
RT-PCR__AtWSD9_R	ATATCGGCTATGGACTGGCTTC
RT-PCR__AtWSD10_F	TACAGAGACGCCTATTAGCGAG
RT-PCR__AtWSD10_R	TGTTCGGTTGAATACCTTAGTTCC
RT-PCR__AtWSD11_F	TTATCCCTAAGAGGTTCGTCCAC
RT-PCR__AtWSD11_R	AAGAACTACACTTGCCTTGAGC
RT-PCR__CsWSD1_F	TGACACTTGTTCTTGCTTGCAC
RT-PCR__CsWSD1_R	CAACGTCGTTCAAGGTCACATTC
RT-PCR__CsWSD2_F	GGAGATATGGAGGAGGAGAGAG
RT-PCR__CsWSD2_R	GGGCAAGGTAGGAAACTTCATG
RT-PCR__CsWSD3_F	TAGGAAAGTTAGTGATGGCGATACG
RT-PCR__CsWSD3_R	TGATCCGAAGCCCTTCTTTAAGAG
RT-PCR__CsWSD4_F	CTAGATCGCCAAATCCTTTACGTG
RT-PCR__CsWSD4_R	AGCTCAACATCTGCATAACAATCG
RT-PCR__CsWSD5_F	TGTTGTCACTGGGATGATCTTC
RT-PCR__CsWSD5_R	CCGTAAGACTCTGTGGAACATTG
RT-PCR__CsWSD6_F	TAAATCAGGAAATGCAAGGACCAC
RT-PCR__CsWSD6_R	GATGGTTACTTTACAGCTCTGTGG

genotyping primers	(5′–3′)
LBb1.3	ATTTTGCCGATTTCGGAAC
wsd1-1_SALK_RP	GATCATATTATTGTGCCGGATCTTG
wsd1-1_SALK_LP	AAGACCTGCTTGTGTCATTCC
AtWSD2-1_CAPS_BtgII_F	ATAAACGTAGAAGAGCATGTCATTG
AtWSD2-1_CAPS_BtgII_R	TATATGAGCTGAGGTGTCACTCG
wsd3-1_dCAPS_TaqaI_F	AATGACACAGGCAGGTCTCTCAAGATATTCG
wsd3-1_dCAPS_TaqaI_R	AACAACATCAAGGTCAATAACACAC
wsd6-2_SALK_RP	AACCGGAAATCGCCTTTGATCG
wsd6-2_SALK_LP	TCGGACAAACGTGGTCGTTAC
fop1-3_wsd11_RP	TTATCCCTAAGAGGTTCGTCCAC
fop1-3_wsd11_LP	GAAACCACACAATCCGTGTAAGAAG
